# The Oral Microbiome–Nitrate–Nitrite–Nitric Oxide Axis and Cardiovascular Health: A Narrative Review

**DOI:** 10.3390/jcm15134871

**Published:** 2026-06-23

**Authors:** Rasha Aziz Attia Salama, Omar Fadi Msalat, Moustafa Medhat Fouad, Mohammed Alhammadi, Siddig Elsheikh, Rashed Ali Nasser

**Affiliations:** 1 Department of Community Medicine, Ras Al Khaimah College of Medicine, Ras Al Khaimah Medical and Health Science University, Ras Al Khaimah 11172, United Arab Emirates; 2 Ras Al Khaimah College of Medicine, Ras Al Khaimah Medical and Health Sciences University, Ras Al Khaimah 11172, United Arab Emirates; omar.24901083@rakmhsu.ac.ae (O.F.M.); moustafa.24901099@rakmhsu.ac.ae (M.M.F.); mohammed.24901010@rakmhsu.ac.ae (M.A.); siddig.24901013@rakmhsu.ac.ae (S.E.); rashed.24901045@rakmhsu.ac.ae (R.A.N.)

**Keywords:** oral microbiome, nitric oxide, nitrate–nitrite pathway, cardiovascular health, endothelial function, blood pressure, nitrate-reducing bacteria, dysbiosis

## Abstract

**Background**: The oral microbiome has emerged as a potential contributor to cardiovascular physiology through its role in the enterosalivary nitrate–nitrite–nitric oxide pathway. Oral nitrate-reducing bacteria convert dietary nitrate into nitrite, which can subsequently be reduced to nitric oxide, a signaling molecule associated with vascular tone, endothelial function, platelet activity, and blood pressure regulation. Disruption of this pathway has been associated with reduced nitric oxide bioavailability and impaired vascular responses. **Methods**: This narrative review summarizes current evidence regarding the relationship between the oral microbiome, nitrate metabolism, and cardiovascular function. Relevant literature was identified through searches of PubMed/MEDLINE and Google Scholar up to May 2026. Evidence from mechanistic, observational, and interventional human studies was reviewed and synthesized thematically. **Results**: Available evidence suggests that oral nitrate-reducing bacteria may influence nitric oxide bioavailability and vascular function. Studies have reported associations between oral microbiome disruption and changes in blood pressure, endothelial responsiveness, plasma nitrite concentrations, and other surrogate cardiovascular markers. However, findings remain heterogeneous and are influenced by factors such as diet, oral hygiene practices, smoking status, medication use, oral health, and underlying cardiometabolic conditions. Most studies are limited by small sample sizes, short intervention durations, and reliance on surrogate outcomes rather than major cardiovascular events. **Conclusions**: The oral microbiome may influence cardiovascular health through its role in nitrate metabolism and nitric oxide bioavailability. However, current evidence is largely limited to surrogate vascular outcomes, while data on major cardiovascular events remain scarce. Further longitudinal and interventional studies are needed to clarify causality and evaluate microbiome-targeted interventions.

## 1. Introduction

The role of the oral microbiome extends beyond oral health to encompass important systemic physiological functions, including cardiometabolic regulation [1,2,3]. The human oral cavity hosts a diverse microbial community involved in metabolic processes that can influence host physiology [4]. One of the most clinically relevant interactions between the oral microbiome and cardiovascular health is the enterosalivary nitrate–nitrite–nitric oxide (NO) pathway [5]. Nitric oxide is a key regulator of vascular tone, endothelial function, platelet aggregation, and cardiovascular homeostasis [6]. Although NO is traditionally generated through the L-arginine–endothelial nitric oxide synthase (eNOS) pathway, an alternative nitrate-dependent pathway relies substantially on oral microbial activity.

Dietary nitrate, primarily obtained from green leafy vegetables and beetroot, is absorbed and concentrated in saliva, where oral bacteria reduce nitrate to nitrite [7,8]. Key nitrate-reducing genera include Rothia, Actinomyces, Veillonella, and Neisseria, which possess nitrate reductase enzymes that facilitate this conversion [9,10]. These microorganisms are particularly abundant on the dorsal tongue surface, where local environmental conditions favor nitrate reduction [10,11]. Following swallowing, nitrite can be further reduced to nitric oxide in the acidic gastric environment or within peripheral tissues under hypoxic conditions, thereby contributing to systemic nitric oxide bioavailability [9]. Because humans lack efficient endogenous nitrate reductase activity, oral microorganisms play an essential role in this pathway.

Growing evidence suggests that disruption of oral microbial ecology through antiseptic mouthwash use, antibiotic exposure, poor oral health, or periodontal disease may impair nitrate reduction and decrease nitric oxide bioavailability [12,13]. Experimental studies have demonstrated that suppression of oral nitrate-reducing bacteria can attenuate nitrate-derived increases in plasma nitrite concentrations and impair blood pressure responses [14]. Conversely, preservation of oral nitrate-reducing capacity may support vascular function, particularly in the context of nitrate-rich dietary patterns. Recent advances in metagenomics, metatranscriptomics, metabolomics, and systems microbiology have further shifted attention from simple taxonomic descriptions toward functional characterization of oral microbial communities and their contribution to nitrate metabolism, nitric oxide generation, and cardiometabolic health [1,2,10,11,15].

Despite increasing interest in this field, important uncertainties remain. Available studies are highly heterogeneous with respect to participant characteristics, oral microbiome assessment methods, dietary nitrate exposure, oral health status, medication use, and outcome measures. Furthermore, most investigations have evaluated surrogate vascular outcomes, such as blood pressure, endothelial function, plasma nitrite concentrations, and vascular responsiveness, rather than major adverse cardiovascular events. Consequently, although biological plausibility is strong, the long-term cardiovascular significance of oral microbiome–mediated nitrate metabolism remains incompletely understood. Therefore, this narrative review aims to synthesize current evidence regarding the role of the oral microbiome in modulating the nitrate–nitrite–nitric oxide pathway and its implications for cardiovascular health, with particular emphasis on mechanistic evidence, vascular physiology, clinical relevance, and current evidence gaps.

## 2. Literature Search Strategy

A narrative review was conducted to synthesize current evidence regarding the relationship between the oral microbiome, the nitrate–nitrite–nitric oxide pathway, and cardiovascular health. A structured literature search was performed using PubMed/MEDLINE and Google Scholar, supplemented by manual screening of reference lists from relevant articles to identify additional studies. Relevant articles published in English up to 31 December 2025 were considered.

The search strategy incorporated combinations of Medical Subject Headings (MeSH) and free-text keywords, including “oral microbiome”, “oral microbiota”, “oral bacteria”, “nitrate”, “nitrite”, “nitrate reductase”, “nitric oxide”, “cardiovascular disease”, “cardiovascular health”, “hypertension”, “blood pressure”, “vascular function”, “endothelial function”, and “dietary nitrate”. Boolean operators (AND, OR) were used to refine the search strategy.

Titles and abstracts identified through the search were screened for relevance, followed by full-text assessment of potentially eligible articles. Studies were considered eligible if they involved adult human participants, examined the oral microbiome in relation to nitrate–nitrite–nitric oxide metabolism, and reported cardiovascular-related outcomes, including blood pressure, endothelial function, vascular responsiveness, exercise performance, or nitric oxide-related biomarkers. Observational, interventional, randomized, cohort, case–control, and cross-sectional studies were eligible for inclusion. Animal and in vitro studies were generally excluded, although selected mechanistic studies were retained where necessary to support biological plausibility and facilitate interpretation of human findings. Reviews, editorials, conference abstracts, and studies lacking relevance to cardiovascular physiology or nitrate metabolism were excluded. Duplicate records identified across databases were removed before screening.

The initial search identified approximately 120 articles. Following title and abstract screening, 45 articles underwent full-text review, and 22 studies were selected for detailed synthesis. As this was a narrative review rather than a systematic review, studies were selected based on methodological quality, clinical relevance, and contribution to understanding the oral microbiome–nitrate–nitrite–nitric oxide axis and cardiovascular physiology. Particular emphasis was placed on human studies evaluating nitrate-reducing oral bacteria, nitric oxide bioavailability, vascular function, blood pressure regulation, and factors influencing oral nitrate metabolism.

To facilitate critical interpretation of the evidence, studies were considered according to their methodological design and level of clinical relevance, including mechanistic investigations, observational studies, human experimental studies, and clinically oriented intervention studies. Evidence was synthesized thematically across four major domains: (1) oral microbiome composition and nitrate-reducing bacteria, (2) the nitrate–nitrite–nitric oxide pathway, (3) cardiovascular implications and vascular function, and (4) factors influencing nitrate metabolism, clinical translation, and future research directions. Findings were interpreted with consideration of methodological strengths, limitations, consistency of findings, and remaining knowledge gaps within the field.

Key human studies were summarized descriptively according to study design, population characteristics, sample size, intervention or exposure, duration where available, nitric oxide-related outcomes, cardiovascular findings, and major methodological limitations, as presented in Table 1.

Systematic reviews and meta-analyses were discussed narratively rather than included in Table 1 because the table was designed to summarize primary human studies evaluating the oral microbiome–nitrate–nitrite–nitric oxide pathway and cardiovascular outcomes.

## 3. Oral Microbiome as a Systemic Modulator

The oral microbiome is increasingly recognized as a functionally active ecosystem with important effects extending beyond oral health to systemic physiological regulation, including cardiometabolic and vascular processes [1,2]. Oral microorganisms contribute to host metabolism through enzymatic activities that humans cannot perform efficiently on their own, rather than serving solely as a passive microbial community. Advances in metagenomic and functional profiling have further demonstrated that microbial metabolic activity may be more relevant to cardiovascular physiology than microbial composition alone [7,10,27].

The reduction of dietary nitrate to nitrite by nitrate-reducing oral bacteria is one of the most clinically relevant examples of this host–microbiome interaction, thereby contributing to systemic nitric oxide bioavailability. Species such as Rothia, Actinomyces, Veillonella, and Neisseria are particularly important because humans lack efficient endogenous nitrate reductase activity [7]. These bacteria are frequently concentrated on the dorsum surface of the tongue, which represents a major site of oral nitrate reduction.

Emerging evidence also suggests that dietary nitrate may exert prebiotic-like effects on the oral microbiome by selectively promoting nitrate-reducing bacterial populations associated with vascular and oral health [8]. This supports a bidirectional relationship in which the oral microbiome facilitates nitrate metabolism while dietary nitrate simultaneously influences microbial composition and metabolic function. Individuals with greater nitrate-reducing capacity have been shown to exhibit higher salivary and plasma nitrite levels, supporting the functional relevance of oral microbial metabolism to systemic nitric oxide production [28].

Two distinct biological processes may disrupt the oral microbiome–nitrate–nitrite–nitric oxide pathway. First, exposure to broad-spectrum antiseptic mouthwashes or antibiotics may reduce the abundance and metabolic activity of nitrate-reducing bacteria through non-selective microbial depletion. This reduction in nitrate-reducing capacity may impair nitrate-to-nitrite conversion and decrease nitric oxide bioavailability, thereby attenuating vascular responses associated with dietary nitrate intake [12,13,22].

In contrast, periodontal disease represents a form of inflammatory dysbiosis characterized by expansion of pathogenic taxa, including Porphyromonas gingivalis and Fusobacterium nucleatum, together with disruption of normal microbial ecological balance [13,29]. Unlike antiseptic-induced microbial depletion, periodontal dysbiosis promotes chronic local and systemic inflammation, oxidative stress, endothelial activation, and vascular dysfunction through immune-mediated mechanisms. Although both conditions may adversely affect cardiovascular physiology, they operate through distinct biological pathways and should not be considered equivalent manifestations of oral dysbiosis.

These alterations may reduce beneficial nitrate-reducing bacteria while increasing pathogenic or pro-inflammatory taxa such as Porphyromonas and Fusobacterium, which are associated with periodontal inflammation and systemic inflammatory burden [13]. This microbial imbalance provides a potential pathway through which oral dysbiosis contributes to endothelial dysfunction, reduced nitric oxide bioavailability, increased blood pressure, and heightened cardiovascular risk.

In addition to impaired nitrate reduction, oral dysbiosis may contribute to systemic cardiovascular effects through chronic inflammatory activation. Periodontal pathogens such as Porphyromonas gingivalis and Fusobacterium nucleatum may promote the release of pro-inflammatory cytokines, including interleukin-6, tumor necrosis factor-α, and C-reactive protein, which are associated with endothelial activation and vascular inflammation. Chronic low-grade inflammation may further impair endothelial nitric oxide synthase activity and exacerbate oxidative stress, thereby linking oral microbial imbalance to both inflammatory and nitric oxide-dependent pathways of cardiovascular injury [13,29,30,31].

Oral nitrate metabolism is also influenced by overall oral ecological balance rather than the presence of individual bacterial taxa alone. The oral microbiome functions as a dynamic microbial ecosystem characterized by complex interspecies interactions, metabolic cooperation, substrate competition, and environmental adaptation. Beneficial nitrate-reducing bacteria may coexist with or be suppressed by pro-inflammatory periodontal pathogens depending on local ecological conditions such as pH, oxygen availability, salivary flow, dietary exposure, oral hygiene practices, and inflammatory status. Consequently, disruption of oral ecological balance may impair nitrate reduction efficiency, alter microbial metabolic activity, and promote inflammatory pathways that contribute to endothelial dysfunction and cardiovascular risk. These findings suggest that microbial functional interactions and oral ecological stability may be more clinically significant than bacterial abundance alone [7,8,10,13,32].

The relationship between oral and cardiovascular health is likely bidirectional. While oral dysbiosis may contribute to endothelial dysfunction, systemic inflammation, and impaired nitric oxide bioavailability, cardiovascular and cardiometabolic diseases may also influence the oral environment through alterations in salivary composition, immune function, medication exposure, and inflammatory status. Consequently, interactions between oral microbial ecology and cardiovascular physiology may be mutually reinforcing rather than unidirectional, highlighting the importance of integrated approaches to oral and systemic health [13,29,30,31].

However, despite strong mechanistic evidence, current findings remain limited by heterogeneity in microbiome assessment methods, dietary nitrate exposure, oral hygiene practices, and cardiovascular outcome measures. In addition, most available studies are short-term and focus on surrogate vascular outcomes rather than long-term cardiovascular events. Therefore, while the oral microbiome–nitric oxide axis is clinically promising, causal relationships and long-term cardiovascular implications remain incompletely established.

## 4. Microbiome Characterization and Functional Assessment

Understanding the relationship between the oral microbiome and nitrate metabolism requires accurate characterization of both microbial composition and function. Several complementary methodologies have been employed to investigate oral microbial communities and their contribution to the nitrate–nitrite–nitric oxide pathway.

The most widely used approach is 16S ribosomal RNA (16S rRNA) gene sequencing, which enables identification and relative quantification of bacterial taxa within oral microbial communities. Using this technique, several nitrate-reducing bacterial taxa previously described have been consistently identified within oral microbial communities and linked to nitrate metabolism [1,2]. However, 16S rRNA sequencing primarily provides taxonomic information and cannot directly assess microbial metabolic activity or nitrate-reducing function.

To overcome these limitations, shotgun metagenomic sequencing has increasingly been utilized. Unlike 16S rRNA profiling, metagenomics enables comprehensive analysis of microbial genes and metabolic pathways, including nitrate reductase enzymes involved in nitrate reduction and nitric oxide generation [33]. These approaches provide greater insight into the functional potential of oral microbial communities and may improve understanding of host–microbe interactions relevant to cardiovascular physiology.

Functional assessment is particularly important because bacterial abundance does not necessarily reflect nitrate-reducing capacity. Consequently, several studies have incorporated measurements of salivary nitrate and nitrite concentrations, nitrate-reduction assays, and nitric oxide metabolites as direct indicators of microbial activity [28,34,35]. Emerging metabolomic and metatranscriptomic approaches further enable evaluation of active microbial pathways and host–microbe metabolic interactions, potentially providing a more clinically meaningful assessment of nitrate-dependent nitric oxide bioavailability [10].

Despite these advances, considerable heterogeneity remains regarding oral sampling techniques, sequencing platforms, bioinformatic pipelines, taxonomic classification methods, and functional assays. Greater methodological standardization is needed to facilitate comparison across studies and improve translation of oral microbiome research into clinical cardiovascular investigations. Because microbial composition does not necessarily reflect metabolic activity, integration of taxonomic, functional, and metabolomic assessments may provide a more comprehensive evaluation of oral nitrate metabolism and nitric oxide bioavailability [10,35,36].

## 5. The Nitrate–Nitrite–Nitric Oxide Pathway

The nitrate–nitrite–nitric oxide pathway represents a microbiome-dependent mechanism that complements the classical L-arginine–endothelial nitric oxide synthase (eNOS) pathway [29,37]. Unlike endogenous nitric oxide synthesis, which is oxygen-dependent and may become impaired in aging, oxidative stress, endothelial dysfunction, hypertension, diabetes, and cardiovascular disease, the nitrate–nitrite pathway provides an alternative source of nitric oxide, particularly under hypoxic or acidic conditions [38].

Dietary nitrate, obtained mainly from green leafy vegetables and beetroot, is rapidly absorbed in the upper gastrointestinal tract and enters the systemic circulation. Approximately 20–25% of circulating nitrate is actively concentrated by the salivary glands and secreted into saliva [30,34]. Within the oral cavity, nitrate-reducing bacteria convert nitrate to nitrite via nitrate reductase activity. Swallowed nitrite can subsequently be reduced to nitric oxide in the acidic gastric environment or in blood and tissues via enzymatic and non-enzymatic pathways [9].

This enterosalivary pathway is critically dependent on oral microbial activity because humans lack efficient endogenous nitrate reductase enzymes. Experimental human studies have demonstrated that suppression of oral bacterial activity using antibacterial mouthwash significantly reduces plasma nitrite generation following dietary nitrate intake [12,16,22]. These findings provide functional evidence that the cardiovascular effects of dietary nitrate depend not only on dietary nitrite intake itself but also on preservation of the oral microbiome.

Nitric oxide generated through this pathway contributes to multiple physiological processes relevant to cardiovascular health, including vasodilation, endothelial protection, platelet inhibition, mitochondrial efficiency, oxygen utilization, and vascular homeostasis [17,18]. Consequently, impaired nitrate reduction caused by oral dysbiosis or disruption of nitrate-reducing bacteria may contribute to endothelial dysfunction and vascular impairment.

Despite growing mechanistic evidence, several uncertainties remain. The efficiency of nitrate reduction appears to vary substantially between individuals and may be influenced by oral hygiene practices, smoking, salivary flow, diet, medication use, periodontal disease, age, and microbiome composition [13,20,39]. Furthermore, bacterial abundance does not necessarily reflect functional nitrate-reducing capacity, highlighting the importance of functional microbiome assessment rather than taxonomic profiling alone.

Therefore, while the nitrate–nitrite–nitric oxide pathway represents a promising link between the oral microbiome and cardiovascular physiology, additional longitudinal and mechanistic studies are required to clarify its long-term clinical significance and therapeutic potential.

**Figure 1:**
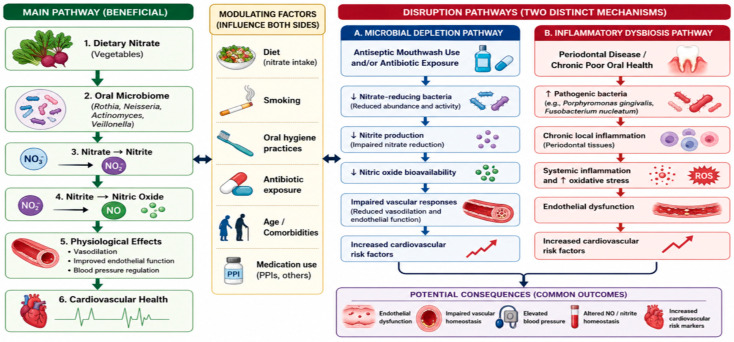
Conceptual framework of the oral microbiome–nitrate–nitrite–nitric oxide pathway and its relationship with cardiovascular health. Dietary nitrate derived from vegetables is converted to nitrite by nitrate-reducing oral bacteria and subsequently contributes to nitric oxide generation, supporting vascular homeostasis through vasodilation, endothelial function, and blood pressure regulation. Two distinct mechanisms may disrupt this pathway. Antiseptic mouthwash use and antibiotic exposure primarily impair nitrate metabolism through depletion of nitrate-reducing bacteria, resulting in reduced nitrite production and nitric oxide bioavailability. In contrast, periodontal disease promotes inflammatory dysbiosis characterized by expansion of pathogenic bacteria, chronic local inflammation, oxidative stress, systemic inflammation, and endothelial dysfunction. Although both pathways may adversely affect vascular physiology and increase cardiovascular risk, they operate through different biological mechanisms and should not be considered equivalent forms of oral dysbiosis. Solid arrows (→) indicate biological pathways or progression of events, bidirectional arrows (↔) indicate modulatory influences, and upward (↑) and downward (↓) symbols denote increases and decreases, respectively. Created by the authors using PowerPoint.

The proposed relationship between the oral microbiome, nitrate metabolism, nitric oxide bioavailability, and cardiovascular risk is illustrated in Figure 1.

In addition to serving as an alternative source of nitric oxide, the nitrate–nitrite–nitric oxide pathway may interact closely with endothelial nitric oxide synthase (eNOS)-dependent signaling and vascular redox homeostasis [29,30]. Under physiological conditions, eNOS generates nitric oxide from L-arginine in an oxygen-dependent reaction that contributes to endothelial function, vascular tone regulation, inhibition of platelet aggregation, and maintenance of vascular integrity [5,6]. However, in cardiovascular disease states, including hypertension, diabetes, atherosclerosis, aging, and chronic inflammation, endothelial nitric oxide production may become impaired due to oxidative stress and eNOS uncoupling.

Oxidative depletion of tetrahydrobiopterin (BH4) or substrate imbalance may lead to eNOS uncoupling, causing eNOS to produce superoxide rather than nitric oxide, thereby contributing to endothelial dysfunction and vascular oxidative injury. Increased reactive oxygen species (ROS) may further reduce nitric oxide bioavailability through the formation of peroxynitrite and other reactive nitrogen species, amplifying vascular inflammation and endothelial impairment [29,30].

Persistent oxidative stress may further exacerbate vascular injury through oxidation of lipids, endothelial DNA damage, mitochondrial dysfunction, and propagation of inflammatory signaling cascades. Formation of peroxynitrite from the interaction between nitric oxide and superoxide may additionally reduce nitric oxide bioavailability while contributing to endothelial cytotoxicity and vascular dysfunction. This redox imbalance represents a central mechanism linking impaired nitric oxide signaling to the progression of cardiovascular disease [29,30,31].

Under these conditions, the nitrate–nitrite–nitric oxide pathway may function as an important compensatory mechanism because nitrite reduction to nitric oxide can occur independently of oxygen availability and eNOS activity, particularly in hypoxic or acidic environments [30,38]. This alternative nitric oxide-generating pathway may therefore partially preserve nitric oxide signaling during impaired endothelial function and vascular oxidative stress.

Nitric oxide generated through nitrate metabolism activates soluble guanylate cyclase and cyclic guanosine monophosphate (cGMP)-dependent signaling pathways, leading to vascular smooth muscle relaxation, improved endothelial responsiveness, and modulation of platelet and inflammatory activity [18,30]. In addition, nitrate-derived nitric oxide may influence mitochondrial respiration and oxygen utilization by modulating components of the electron transport chain, thereby improving mitochondrial efficiency and reducing oxidative stress [18].

Emerging evidence also suggests that oral microbial functional activity may influence broader cardiometabolic signaling pathways involving endothelial inflammation, insulin resistance, and vascular redox balance. However, the precise molecular interactions between oral microbial nitrate metabolism, endothelial nitric oxide synthase activity, reactive oxygen species generation, and cardiovascular disease progression remain incompletely understood.

## 6. Impact on Cardiovascular Function

Nitric oxide is a crucial regulator of cardiovascular physiology, contributing to vasodilation, endothelial homeostasis, inhibition of platelet aggregation, modulation of vascular inflammation, and maintenance of blood pressure regulation [30]. Reduced nitric oxide bioavailability has been strongly associated with endothelial dysfunction, hypertension, arterial stiffness, atherosclerosis, and adverse cardiovascular outcomes [31].

At the molecular level, nitric oxide exerts many of its vascular effects through activation of soluble guanylate cyclase and subsequent cyclic guanosine monophosphate (cGMP)-mediated signaling pathways, which promote vascular smooth muscle relaxation and endothelial homeostasis [30]. Nitric oxide also modulates mitochondrial respiration, leukocyte adhesion, platelet activation, and vascular inflammatory signaling. Consequently, reduced nitric oxide bioavailability may contribute not only to impaired vasodilation but also to oxidative stress, vascular inflammation, arterial stiffness, and progression of atherosclerotic vascular disease.

Reduced nitric oxide signaling impairs endothelium-dependent vasodilation and promotes vascular smooth muscle constriction, increased leukocyte adhesion, platelet activation, and pro-inflammatory endothelial signaling. In addition, reduced nitric oxide availability may enhance oxidative stress through an imbalance between nitric oxide and reactive oxygen species generation, thereby promoting vascular dysfunction and atherosclerotic progression. These processes are particularly relevant in hypertension, diabetes, metabolic syndrome, and aging, where endothelial dysfunction is considered an early marker of cardiovascular disease development [29,30,31].

Clinical and experimental studies suggest that activation of the nitrate–nitrite–nitric oxide pathway through dietary nitrate supplementation may improve vascular function and reduce blood pressure in both healthy individuals and populations at increased cardiovascular risk [17,40,41]. Acute dietary nitrate intake has been associated with improvements in endothelial function, vasodilation, platelet regulation, and vascular responsiveness, supporting the physiological relevance of nitrate-derived nitric oxide to cardiovascular homeostasis [17].

In individuals with hypertension, type 2 diabetes, peripheral arterial disease, and hypercholesterolemia, dietary nitrate supplementation has demonstrated potential benefits, including reductions in systolic blood pressure, improved endothelial responsiveness, and enhanced exercise capacity [32,36,37,38,39]. These observations are particularly relevant because endogenous nitric oxide production is frequently impaired in cardiometabolic disease, suggesting that the enterosalivary nitrate pathway may partially compensate for reduced endothelial nitric oxide synthase activity.

Importantly, disruption of oral microbial nitrate metabolism has been associated with reduced nitrite generation and attenuation of nitrate-mediated vascular responses [16,22,24]. These findings provide functional evidence that oral bacteria contribute directly to nitrate bioactivation and nitric oxide generation. Beyond blood pressure regulation, nitrate-derived nitric oxide may influence vascular compliance, mitochondrial efficiency, oxygen utilization, and inflammatory signaling pathways, supporting a broader role in cardiovascular physiology [17,18,30].

Although studies involving healthy volunteers have provided important mechanistic insights, evidence from populations with established cardiovascular and cardiometabolic risk factors is particularly relevant when evaluating potential clinical significance. Nevertheless, the current evidence base remains heterogeneous and not universally consistent. While many studies report favorable associations between nitrate-reducing oral bacteria, nitric oxide bioavailability, and vascular function, other investigations have demonstrated weaker or inconsistent relationships. Variability in study populations, oral health status, dietary nitrate exposure, medication use, microbiome assessment methodologies, and cardiovascular outcome definitions may contribute to these discrepancies.

Furthermore, bacterial abundance alone may not accurately reflect functional nitrate-reducing activity, and improvements in surrogate vascular markers such as blood pressure, plasma nitrite concentrations, endothelial function, and exercise capacity do not necessarily translate into reductions in myocardial infarction, stroke, heart failure, cardiovascular mortality, or other major adverse cardiovascular events. Consequently, although the biological plausibility of the oral microbiome–nitrate–nitrite–nitric oxide axis is well supported, its long-term clinical significance remains uncertain.

Recent advances in multi-omics technologies, including metagenomics, metatranscriptomics, metabolomics, and systems microbiology, have shifted the field from predominantly taxonomic descriptions of oral microbial communities toward functional characterization of microbial nitrate-reduction capacity and host–microbiome interactions [25,39,42,43,44]. These approaches enable direct assessment of microbial metabolic pathways, functional gene expression, microbial metabolite production, and their relationship to nitric oxide bioavailability, vascular function, and cardiometabolic health. Consequently, they may provide a more comprehensive understanding of the biological mechanisms linking oral microbial ecology with cardiovascular physiology than taxonomic profiling alone. However, integration of these methodologies into large-scale longitudinal and interventional studies remains limited, and their clinical applicability requires further validation.

## 7. Modulating Factors

The efficiency of the oral microbiome–nitrate–nitrite–nitric oxide pathway is influenced by numerous microbial, environmental, dietary, behavioral, and host-related factors that may either enhance or impair nitrate reduction and nitric oxide bioavailability. Understanding these factors is important for interpreting interindividual variability in vascular responses to dietary nitrate and for identifying potential targets for intervention.

Oral hygiene practices are important modifiers of nitrate metabolism. Antiseptic mouthwash use and periodontal disease remain important modifiers of nitrate metabolism and oral microbial ecology and should be considered when interpreting interindividual variability in vascular responses. Dietary nitrate intake is another major determinant of pathway activity. Nitrate-rich foods, particularly beetroot and green leafy vegetables, have consistently been associated with improvements in endothelial function, vascular responsiveness, and blood pressure regulation [37,39,40]. However, these foods also contain other bioactive compounds, including potassium, vitamin C, polyphenols, and dietary fiber, which may independently contribute to cardiovascular health. Consequently, the vascular benefits associated with nitrate-rich foods cannot be attributed exclusively to nitrate metabolism, and the relative contribution of nitrate versus other nutritional components remains incompletely understood.

Several host-related factors may further influence nitrate metabolism and contribute to variability in nitric oxide bioavailability. Age, salivary flow, smoking, oral hygiene status, periodontal disease, medication use, gastric acidity, and underlying cardiometabolic conditions may all affect nitrate reduction efficiency [13,20,39]. Smoking may alter oral microbial ecology through oxidative stress, reduced salivary flow, and promotion of periodontal pathogens, thereby impairing nitrate-reducing capacity [13,39]. Antibiotic exposure can transiently suppress nitrate-reducing bacterial populations and reduce nitrate bioactivation.

Proton pump inhibitors may represent a particularly important modifier of the enterosalivary nitrate–nitrite–nitric oxide pathway. Gastric acidity plays a critical role in the chemical reduction of swallowed nitrite to nitric oxide within the stomach [9,30]. Consequently, chronic acid suppression and pharmacological achlorhydria may substantially impair nitric oxide generation and potentially attenuate, or in some individuals even abolish, vascular benefits associated with dietary nitrate supplementation [30,39]. Although the clinical significance of this interaction remains incompletely understood, proton pump inhibitor use should be considered an important confounding factor in studies evaluating nitrate metabolism, nitric oxide bioavailability, and cardiovascular responses [10,30,39].

Collectively, these findings indicate that the cardiovascular effects of dietary nitrate are influenced not only by nitrate intake itself but also by preservation of oral microbial function, ecological stability, host physiology, and environmental exposures. These interacting factors likely contribute to the heterogeneity observed across studies evaluating the oral microbiome–nitrate–nitrite–nitric oxide axis and cardiovascular health.

## 8. Safety Considerations

Although dietary nitrate supplementation is generally considered safe at doses commonly consumed through vegetables and beetroot products, concerns have historically been raised regarding nitrosamine formation and methemoglobinemia [11,43]. In particular, N-nitrosamines, a class of compounds with recognized carcinogenic potential, may theoretically form through reactions between nitrite and amines under acidic gastric conditions [11]. However, concerns regarding nitrate exposure have largely originated from processed and preserved foods rather than nitrate-rich vegetables. Importantly, vegetables contain substantial amounts of antioxidants, including vitamin C and polyphenols, which may inhibit endogenous nitrosamine formation and mitigate potential carcinogenic effects [11,44].

Current evidence suggests that nitrate-rich vegetables and dietary nitrate supplementation within recommended dietary ranges are generally safe and may provide cardiovascular and metabolic benefits [15,44]. Nevertheless, several factors may influence nitrate metabolism and nitric oxide bioavailability, including gastric acidity, medication use, and underlying medical conditions. For example, chronic proton pump inhibitor use may impair gastric nitrite-to-nitric oxide conversion by reducing gastric acidity, potentially altering both the efficacy and physiological consequences of nitrate supplementation [30,39].

Clinical and experimental studies have generally demonstrated favorable safety profiles for dietary nitrate supplementation when consumed within recommended dietary ranges [15,44]. However, long-term safety data remain limited, particularly among individuals with impaired nitrate metabolism, severe renal dysfunction, chronic acid suppression therapy, or conditions predisposing to methemoglobinemia [15,43]. Therefore, although current evidence supports the overall safety of dietary nitrate derived from vegetables, additional long-term studies are required to establish safety profiles across diverse patient populations and to better characterize potential risks associated with chronic supplementation.

## 9. Clinical Implications

The oral microbiome may represent a modifiable contributor to cardiovascular health through its role in nitrate metabolism and nitric oxide bioavailability [6]. Nitrate-reducing oral bacteria support nitric oxide generation, thereby contributing to vascular tone regulation, endothelial function, and blood pressure homeostasis [31,45]. Preservation of a functionally balanced oral microbiome may therefore be relevant to vascular health maintenance.

Practical strategies that may support the nitrate–nitrite–nitric oxide pathway include consumption of nitrate-rich vegetables, maintenance of good oral health, prevention and treatment of periodontal disease, and avoidance of unnecessary disruption of nitrate-reducing oral bacteria through excessive use of antiseptic mouthwashes or inappropriate antibiotic exposure [10,11,15,30,43,44]. Dietary nitrate intake from beetroot, spinach, lettuce, and other leafy vegetables has consistently been associated with improved vascular responses and blood pressure regulation, although these effects appear to depend largely on preservation of oral microbial nitrate-reducing activity [37,39,41,45,46,47].

Emerging microbiome-targeted approaches, including nitrate-rich diets, prebiotic strategies, and nitrate-reducing probiotics, represent promising areas for future cardiovascular research [8,41]. However, current evidence primarily supports mechanistic, observational, and short-term improvements in surrogate vascular outcomes, including blood pressure, endothelial function, plasma nitrite concentrations, and vascular responsiveness, rather than confirmed reductions in cardiovascular events or mortality. Importantly, current evidence does not establish a direct causal relationship between oral microbiome alterations and cardiovascular disease. Therefore, the oral microbiome–nitrate–nitrite–nitric oxide axis should currently be viewed as a promising but incompletely validated target for cardiovascular prevention and intervention.

Additional longitudinal and randomized interventional studies evaluating clinically meaningful cardiovascular outcomes are required before microbiome-targeted strategies can be translated into routine clinical practice.

Key clinical implications include:

1. The oral microbiome contributes to nitric oxide-mediated vascular regulation [4,21,26].
2. Disruption of nitrate-reducing oral bacteria may impair nitrate bioactivation and vascular responses [12,16,22].
3. Nitrate-rich diets may support vascular health when oral microbial nitrate-reducing capacity is preserved [37,39,41,45,46,47].
4. Excessive use of broad-spectrum antiseptic mouthwash may reduce nitric oxide bioavailability [12,16,22].
5. Current evidence remains insufficient to support formal cardiovascular guideline recommendations related to oral microbiome modulation [24,26,36].

The major clinical implications, current evidence, and remaining research gaps related to the oral microbiome–nitric oxide–cardiovascular axis are summarized in Table 2.

## 10. Limitations

Several important limitations should be acknowledged. First, as a narrative review, the study selection process was not based on a formal systematic review methodology, which may introduce selection bias. Formal risk-of-bias assessment tools were not applied; instead, studies were selected based on methodological quality, clinical relevance, and their contribution to understanding the oral microbiome–nitrate–nitrite–nitric oxide axis and cardiovascular health.

Second, the literature search was restricted to PubMed/MEDLINE and Google Scholar, supplemented by manual screening of reference lists. Although these databases capture a substantial proportion of the biomedical literature, potentially relevant studies indexed exclusively in other databases, including Embase, Scopus, Web of Science, and the Cochrane Library, may not have been identified. Consequently, some degree of publication and database selection bias cannot be excluded.

The available literature remains highly heterogeneous with respect to study design, participant characteristics, oral microbiome assessment techniques, nitrate supplementation protocols, oral hygiene exposures, and cardiovascular outcome measures, limiting direct comparison across studies. A recent systematic review by Puel et al. reported inconsistent associations between nitrate-reducing oral bacteria and hypertension across observational studies, highlighting substantial variability in microbial assessment methods, participant characteristics, and cardiovascular outcomes [36].

Most available studies involve relatively small sample sizes, short intervention durations, and reliance on surrogate vascular outcomes, including blood pressure, endothelial function, plasma nitrite concentrations, and vascular responsiveness, rather than major adverse cardiovascular events. Consequently, evidence linking oral microbiome modulation to reductions in myocardial infarction, stroke, heart failure, cardiovascular mortality, or other clinically meaningful cardiovascular outcomes remains limited.

Interpretation of dietary nitrate intervention studies may also be influenced by methodological challenges related to placebo design and participant blinding. Although several randomized trials included nitrate-depleted beetroot juice or similar placebo preparations to reduce expectation bias, complete blinding may be difficult because taste, color, and other sensory characteristics can differ between interventions. Consequently, placebo effects and participant expectations cannot be entirely excluded and may contribute to variability in reported vascular responses.

Interpretation of dietary nitrate intervention studies is further complicated by potential confounding from other bioactive constituents present in nitrate-rich foods, including potassium, vitamin C, polyphenols, and dietary fiber, which may independently contribute to vascular health. Additionally, placebo design and participant blinding may be challenging in nutritional intervention studies, potentially introducing expectation bias.

Several additional factors, including smoking, oral hygiene practices, periodontal disease, medication use, salivary flow, age, gastric acidity, and comorbid conditions, may independently influence both oral microbiome composition and cardiovascular physiology, thereby complicating causal interpretation. Furthermore, bacterial abundance alone may not accurately reflect nitrate-reducing activity or nitric oxide bioavailability, emphasizing the importance of functional microbiome assessment, metabolomics, meta transcriptomics, and standardized nitric oxide-related biomarkers.

Therefore, although current evidence supports the biological plausibility and short-term vascular effects of the oral microbiome–nitrate–nitrite–nitric oxide axis, direct causal relationships between oral microbiome alterations and long-term cardiovascular outcomes remain incompletely established. Larger longitudinal studies and adequately powered randomized interventional trials using standardized methodologies and clinically meaningful cardiovascular endpoints are required before definitive clinical conclusions can be drawn.

## 11. Future Directions

Future research should focus on large-scale longitudinal studies and adequately powered randomized trials to determine whether oral microbiome-mediated nitrate metabolism influences long-term cardiovascular outcomes. Although current evidence supports beneficial effects on surrogate vascular markers, including blood pressure, endothelial function, plasma nitrite concentrations, and vascular responsiveness, evidence linking the oral microbiome–nitrate–nitrite–nitric oxide axis to reductions in myocardial infarction, stroke, heart failure, major adverse cardiovascular events, or cardiovascular mortality remains limited [16,21,46]. Establishing such relationships will require long-term studies incorporating clinically meaningful cardiovascular endpoints.

Greater methodological standardization is needed regarding microbiome characterization, nitrate supplementation protocols, oral hygiene exposure, and cardiovascular outcome assessment. Future investigations should integrate advanced multi-omics approaches, including metagenomics, metatranscriptomics, metabolomics, and functional nitrate-reduction assays, together with measurements of nitrate/nitrite metabolism, nitric oxide bioavailability, and vascular biomarkers [1,2,10,33,35]. Simultaneous assessment of microbial composition, microbial function, host physiology, and cardiovascular outcomes will be essential for clarifying causal mechanisms [2,10,35].

Future studies should also account for key modifiers of nitrate metabolism, including oral health status, periodontal disease, smoking, medication use, gastric acid suppression therapy, age, and cardiometabolic risk factors. Particular attention should be directed toward populations with hypertension, type 2 diabetes, endothelial dysfunction, chronic periodontal disease, impaired salivary flow, and age-related vascular dysfunction, who may derive the greatest benefit from microbiome-targeted interventions [1,10,30,36].

Importantly, emerging therapeutic strategies should be guided by ecological principles. Current evidence suggests that nitrate metabolism depends on the functional stability of complex oral microbial communities rather than on individual bacterial species alone [1,2,36]. Consequently, future interventions should prioritize preservation or restoration of oral microbial ecological balance and nitrate-reducing capacity through ecosystem-based approaches, including dietary nitrate modulation, prebiotic nutritional strategies, optimization of oral hygiene practices, maintenance of oral microbial diversity, and precision nutrition interventions tailored to individual microbial and metabolic profiles [1,2,10,39,43,44]. Although nitrate-reducing probiotic strategies remain of interest, their clinical value should be evaluated within the broader context of oral microbial ecology and community-level function [1,2,13,33,35].

Advances in systems biology, machine-learning approaches, and multi-omics technologies may further improve understanding of the complex interactions between oral microbial ecology, nitrate metabolism, host physiology, and cardiovascular health [10,33,35]. Continued collaboration among oral health researchers, cardiovascular scientists, nutrition specialists, and microbiome investigators will be essential to translate mechanistic insights into safe, evidence-based strategies for improving cardiovascular health through modulation of the oral microbiome.

## 12. Conclusions

This narrative review highlights the important role of the oral microbiome in nitric oxide-mediated cardiovascular regulation through the enterosalivary nitrate–nitrite–nitric oxide pathway. Nitrate-reducing oral bacteria contribute to the conversion of dietary nitrate into nitrite, thereby supporting nitric oxide bioavailability, vascular homeostasis, endothelial function, and blood pressure regulation.

Current mechanistic and short-term clinical evidence suggests that disruption of oral microbial ecology through antiseptic mouthwash use, antibiotic exposure, periodontal disease, poor oral health, or other factors may impair nitrate reduction and reduce nitric oxide bioavailability, potentially contributing to adverse vascular effects. Conversely, preservation of oral microbial nitrate-reducing capacity may support vascular function, particularly in the context of nitrate-rich dietary patterns.

However, despite strong biological plausibility and promising physiological findings, the available evidence remains limited by methodological heterogeneity, relatively small sample sizes, short follow-up periods, and reliance on surrogate vascular outcomes, including blood pressure, endothelial function, plasma nitrite concentrations, and vascular responsiveness. Although evidence supporting effects on these surrogate markers is increasingly robust, direct evidence linking oral microbiome modulation to reductions in myocardial infarction, stroke, heart failure, cardiovascular mortality, or other major adverse cardiovascular events remains limited. Consequently, the extent to which alterations in the oral microbiome influence long-term cardiovascular risk and clinical outcomes remains uncertain.

Collectively, the oral microbiome–nitrate–nitrite–nitric oxide axis represents an emerging area of cardiovascular and microbiome research with potential clinical significance. Further longitudinal and interventional studies using standardized methodologies, functional microbiome assessments, and clinically meaningful cardiovascular endpoints are needed to clarify causality, identify high-risk populations, and evaluate the therapeutic potential of microbiome-targeted cardiovascular interventions.

## Figures and Tables

**Table 1:** Summary of Primary Human Studies Evaluating the Oral Microbiome–Nitrate–Nitrite–Nitric Oxide Pathway and Cardiovascular Outcomes.

Study	Design	Population (n)	Nitrate Source/Intervention	Dose	Duration	Primary Outcome	Main Findings	Major Limitation
**Interventional Studies**								
Govoni et al., 2008 [16]	Experimental human study	Healthy adults (*n* = 10)	Sodium nitrate + antibacterial mouthwash	10 mg/kg sodium nitrate	Single dose	Plasma nitrite concentration	Mouthwash markedly reduced nitrite formation following nitrate ingestion	Small sample size
Webb et al., 2008 [17]	Clinical trial	Healthy adults (*n* = 14)	Beetroot juice	500 mL containing 5.5 mmol nitrate	Single dose	Blood pressure, endothelial function	Improved endothelial function, vasodilation, and blood pressure responses	Acute intervention design
Larsen et al., 2011 [18]	Randomized crossover trial	Healthy adults (*n* = 17)	Sodium nitrate supplementation	0.1 mmol/kg/day	3 days	Blood pressure	Significant reduction in blood pressure	Short intervention duration
Kenjale et al., 2011 [19]	Clinical trial	Peripheral arterial disease patients (*n* = 8)	Beetroot juice	500 mL containing ~9 mmol nitrate	Single dose	Exercise capacity, vascular response	Improved exercise performance and vascular function	Small sample size
Gilchrist et al., 2013 [20]	Clinical trial	Patients with type 2 diabetes (*n* = 27)	Beetroot juice	250 mL containing 7.5 mmol nitrate/day	2 weeks	Endothelial function, insulin sensitivity	Improved endothelial responsiveness	Short intervention period
Kapil et al., 2013 [21]	Randomized crossover trial	Healthy adults (*n* = 19)	Chlorhexidine mouthwash	No nitrate administered	7 days	Blood pressure, plasma nitrite	Reduced nitrite levels and increased blood pressure	Small sample size
Bondonno et al., 2015 [22]	Clinical study	Treated hypertensive adults (*n* = 15)	Antibacterial mouthwash	No nitrate administered	3 days	Blood pressure response	Mouthwash attenuated nitrate-mediated blood pressure reduction	Short duration
Kapil et al., 2015 [23]	Randomized placebo-controlled trial	Hypertensive patients (*n* = 68)	Beetroot juice	250 mL containing 6.4 mmol nitrate/day	4 weeks	Blood pressure	Sustained reduction in blood pressure	Moderate sample size
Velmurugan et al., 2016 [24]	Randomized placebo-controlled trial	Hypercholesterolemic patients (*n* = 69)	Potassium nitrate supplementation	6 mmol/day	6 weeks	Endothelial function, vascular stiffness	Improved vascular responsiveness and endothelial function	Limited long-term follow-up
**Mechanistic/Microbiome Studies**								
Hyde et al., 2014 [7]	Metagenomic study	Human oral samples (*n* = 123)	Shotgun metagenomic sequencing	Observational	Cross-sectional	Nitrate-reducing bacterial taxa	Identified key nitrate-reducing genera including *Rothia*, *Neisseria*, and *Actinomyces*	No cardiovascular outcomes assessed
Bescos et al., 2020 [25]	Experimental microbiome study	Healthy adults (*n* = 36)	Oral microbiome profiling	Chlorhexidine mouthwash	7 days	Oral microbiome composition, nitrite metabolism	Mouthwash altered microbiome composition and nitrate metabolism	No cardiovascular endpoints
**Observational Studies**								
Goh et al., 2022 [26]	Cross-sectional observational study	ORIGINS cohort adults (*n* = 287)	Oral microbiome functional profiling	Cross-sectional	Cardiometabolic risk markers	Nitrite-generating capacity associated with favorable cardiometabolic profiles	Observational design; causal relationships cannot be established	

Note: Considerable heterogeneity exists across studies with respect to nitrate source, dose, duration, oral microbiome assessment techniques, participant characteristics, and cardiovascular outcome measures. Most available studies are short-term and primarily evaluate surrogate vascular outcomes, including blood pressure, endothelial function, plasma nitrite concentrations, and vascular responsiveness, rather than major adverse cardiovascular events.

**Table 2:** Summary of Current Evidence, Strength of Evidence, and Research Gaps Related to the Oral Microbiome–Nitrate–Nitrite–Nitric Oxide Axis.

Topic/Mechanism	Evidence Type	Current Evidence	Strength of Evidence	Clinical Relevance	Evidence Gaps/Future Research Needs	Key References
Oral nitrate-reducing bacteria and nitrite generation	Mechanistic and human experimental studies	Consistent evidence supports a role for oral nitrate-reducing bacteria in nitrate-to-nitrite conversion and nitric oxide bioavailability	Strong	Established physiological relevance; clinical applications remain investigational	Need for standardized functional assessment methods	[8,9,30,37]
Oral microbiome composition and blood pressure regulation	Observational and interventional studies	Associations observed between oral microbiome alterations, nitrate metabolism, and blood pressure responses	Moderate	Potential contributor to cardiovascular risk; not currently a validated clinical target	Limited long-term intervention studies and causal evidence	[17,32,38,40]
Antibacterial mouthwash use and cardiovascular effects	Human experimental and clinical studies	Repeated mouthwash use can impair nitrate reduction and attenuate blood pressure benefits associated with nitrate metabolism	Moderate	Supports caution in interpreting effects on nitrate metabolism; no formal clinical recommendations	Need for larger and longer-duration studies	[13,16,22,24]
Oral microbiome functions as a cardiovascular biomarker	Primarily observational studies	Preliminary associations reported between nitrate-reducing capacity and cardiometabolic health markers	Limited	Potential future biomarker application; not clinically validated	Lack of standardized assays, validation cohorts, and prospective studies	[27,32,36]
Personalized microbiome-based cardiovascular prevention	Hypothesis-generating and early translational research	Concept supported by biological plausibility but with limited direct clinical evidence	Limited	Future research direction rather than current clinical practice	Need for randomized trials and validated intervention strategies	[8,27,32,41]

## Data Availability

No new data were created or analyzed in this study. Data sharing does not apply to this article.

## Abbreviations

The following abbreviations are used in this manuscript:

NO	Nitric Oxide
eNOS	Endothelial Nitric Oxide Synthase
MeSH	Medical Subject Headings
RCT	Randomized Controlled Trial
BP	Blood Pressure
CVD	Cardiovascular Disease
PPI	Proton Pump Inhibitor
NOS	Nitric Oxide Synthase
L-arginine	L-arginine amino acid pathway substrate
NO_3_^−^	Nitrate
NO_2_^−^	Nitrite
